# Prevalence of Hypomagnesaemia among Obese Type 2 Diabetic Patients Attending the National Center for Diabetes, Endocrinology and Genetics (NCDEG)

**DOI:** 10.5812/ijem.17796

**Published:** 2014-07-01

**Authors:** Dana Hyassat, Ebtihaj Al Sitri, Anwar Batieha, Mohammed EL-Khateeb, Kamel Ajlouni

**Affiliations:** 1 The National Center for Diabetes, Endocrinology and Genetics Center, Amman, Jordan; 2 Jordan University of Science and Technology (JUST), Amman, Jordan

**Keywords:** Diabetes Mellitus, Type 2, Jordan, Hypomagnesaemia

## Abstract

**Background:**

Some observations suggested that magnesium supplementation could be helpful in the treatment of diabetic patients by improving glycemic control and preventing the development of diabetes-related complications.

**Objectives:**

To estimate the prevalence of hypomagnesaemia among obese patients with type 2 diabetes attending the National Center for Diabetes, Endocrinology and Genetics (NCDEG) in Amman, Jordan.

**Patients and Methods:**

A cross-sectional study was carried out at the National Center for diabetes, Endocrinology and Genetics (NCDEG) in Amman-Jordan. A total of 1105 patients with type 2 diabetes (51.9% females and 48.1% males) who attended this center between first of October 2011and end of February 2012 were included in the study. The mean age and duration of diabetes were 57.1 years and 5.1 years, respectively and the mean value of HbA1c was 7.9%. Our study also performed a comparison of the prevalence of hypomagnesaemia between our studied sample and 3600 individuals enrolled in the National Vitamin D study completed in Jordan in 2009. The obtained data included patients’ age, gender, smoking history, HbA1c level, comorbid history including hypertension, dyslipidemia, and presence of neuropathy and retinopathy.

**Results:**

Out of 1105 patients with type 2 diabetes, 210 patients (19%) (95% CI, 16.8%-21.4%) were hypomagnesaemic. Female gender, hypertension, statin therapy, HbA1c between 7-7.9% or ≥ 9% and patients with diabetes duration more than five years were independent risk factors for hypomagnesaemia. No association between hypomagnesaemia and age distribution, smoking history, neuropathy and retinopathy was found. In comparison with individuals enrolled in the National Vitamin D study, diabetic patients in this study had a much higher prevalence of hypomagnesaemia (19% vs. 0.7%) with odd’s ratio of 32 (95% CI, 21-48.2).

**Conclusions:**

As the prevalence of hypomagnesaemia among patients with type 2 diabetes treated at the NCDEG was found to be 19% (95% CI, 16.8%-21.4%), we recommend periodic determination of magnesium level and appropriate magnesium replacement therapy particularly among the above defined groups.

## 1. Background

The cation Mg^++^ is the fourth most abundant extracellular and the second most prevalent intracellular cation in the human body (1). Mg^++^ is involved in more than 300 essential and fundamental metabolic reactions (2), including hormone receptor binding, gating of calcium channels, second messenger system, transmembrane ion flux, regulation of muscle contraction and vascular tone, cardiac excitability and neurotransmitter release (3-5).

Magnesium has a fundamental role in carbohydrate metabolism since it is involved at multiple levels in the secretion of insulin and its receptor binding and activity. Diabetes mellitus has been suggested to be the most common metabolic disease associated with magnesium deficiency, having 25 to 39% prevalence (6). The mechanism responsible for hypomagnesaemia in diabetic patients is not fully understood. Osmotic diuresis obviously accounts for a portion of magnesium loss, however magnesium intake may also play a role in magnesium deficiency as individuals do not consume the fully–recommended daily allowance for magnesium. A suppressed level of intercellular magnesium has been reported in diabetic patients. Additionally, hyperinsulinemia in patients with insulin resistance might contribute to magnesium depletion. Glycemic control in type 2 diabetic patients may not correct hypomagnesaemia, suggesting that magnesium levels in diabetic patients could be regulated by other unknown factors (7, 8).

Recently, a great body of evidence has emerged regarding the contribution of magnesium deficiency in the pathogenesis of diabetic complications and is regarded as both a cause and a consequence of these complications. Association between hypomagnesaemia and illnesses such as insulin resistance, dyslipidemia, atherosclerosis, hypertension and unpleasant outcomes in pregnancies have been reported (8, 9). Some observations suggested that magnesium supplementation could be helpful in the treatment of diabetic patients by improving glycemic control and preventing the development of diabetes-related complications (9, 10).

## 2. Objectives

The objectives of the current study were to assess the prevalence of hypomagnesaemia in obese type 2 diabetic patients and to examine the correlation between magnesium and glycemic control.

## 3. Patients and Methods

### 3.1. Sampling and Data Collection

A cross-sectional study was carried out at the National Centre for Diabetes, Endocrinology and Genetics (NCDEG) in Amman-Jordan, which treats patients with Diabetes Mellitus from all over the country. All patients with type 2 diabetes who referred tothe NCDEG during the period from the first of October 2011 to the end of January 2012 and had their serum Mg assessed were included in the study. Our study also included a comparison in the prevalence of hypomagnesaemia between our studied patients and 3600 individuals enrolled in the National Vitamin D study completed in 2009 in Jordan. Patients with the following conditions were excluded from the study: pregnancy, hyperthyroidism, hypoparathyroidism, celiac disease, renal failure, chronic use of steroid medication, antacids and purgatives. Data was obtained from the medical records, which were abstracted by the investigator using a data sheet prepared for the purpose of this study.

The National Vitamin D study was conducted by the NCDEG in 2009 and the raw data were available to the researchers for the purpose of comparison. Magnesium level was measured in that study in addition to a wide array of laboratory measurements. The detailed sample recruitment and methodology of the National Vitamin D Study has been published elsewhere (11). The present study was approved by the National Centre for Diabetes, Endocrinology and Genetics (NCDG) Ethics’ Committee. Identifying information was kept strictly confidential and the data were used only for scientific purposes by the researchers.

### 3.2. Definitions OF the Study Variables

#### 3.2.1. Measurements and Laboratory Analysis

Anthropometric measurements, including weight, height, and waist circumference were measured while the subjects were wearing light clothing and no shoes. Waist circumference was estimated at the end of a normal expiration using a non-strechable tape held in a horizontal plane around the abdomen at the level of the iliac crest. It was considered normal if the waist was between 88.5 to 91.8 cm in men and from 84.5 to 88.5 cm in women, this was according to the anthropometric cutoff values for detecting metabolic abnormalities in Jordanian adults (11). Waist to height ratio was considered normal if waist to height ratio was ≤ 0.5, and elevated if it was > 0.5.

BMI was expressed as the quotient between weight (kg) and height squared (m^2^). Patients were classified according to BMI following the recommendation of the World Health Organization as adopted by the American Diabetes Association (12). Readings of systolic and diastolic blood pressures were taken while the subjects were seated and the arm was kept at the heart level, after at least five minutes of rest, using a standardized mercury sphygmomanometer, high blood pressure was defined as systolic blood pressure ≥ 130 mmHg or diastolic blood pressure ≥ 80 mmHg or if the patient was already on antihypertensive drugs (13).

Metabolic abnormalities were defined according to the American Diabetes Association 2011 (13) as follows: total serum cholesterol ≥200 mg/dL, serum LDL ≥ 100mg/dL, serum triglyceride ≥ 150 mg/dL, serum HDL ≤ 40 mg/dL in men, and ≤ 50 mg/dL in women, or if the patient was already on antidyslipidemic agents. Smoking was classified into three categories according to WHO guidelines 1998; current smoker was defined as: a person who smokes cigarettes daily or occasionally; past-smoker: a person who formerly was a daily or occasional smoker, but currently does not smoke at all; non smoker: a person who has never smoked before or has smoked very little in the past (14).

Diabetes mellitus was diagnosed if the patient had a FPG ≥ 126 mg/dL (7.0 mmol/L) in two occasions or if the patient had a random plasma glucose ≥ 200 mg/dL (11.1 mmol/L) in the presence of classical symptoms of hyperglycemia, or if he or she had HbA1c ≥ 6.5%. Moreover, diabetes was considered to be controlled if the patient had HbA1c < 7.0% according to the American Diabetes Association (ADA) 2011 guidelines (13).

Magnesium was measured by “Colorimetric Endpoint Method” using Roche/Cobas Integra 800 automated system. The imprecision, with run (CV) was 2.8 and 2.9% between runs as judged by internal quality- control systems. Our operational definition of hypomagnesaemia was the occurrence of magnesium level below 1.7 mg/dL (normal range 1.7-2.55 mg/dL). This normal range of magnesium (1.7-2.55 mg/dL) is the normal value provided by our laboratory as there is no published data regarding the normal magnesium values for Jordanian yet.

Retinopathy was diagnosed if it was documented by either the ophthalmologist or the treating physician in the medical records, or if the patient had received laser treatment whereas neuropathy was diagnosed if there was any of the following symptoms (numbness, tingling, or pain in toes, feet, legs, hands, arms, and fingers) in the patient’s medical records or if the patient had done Nerve Conduction Study (NCS) which proves the presence of diabetic neuropathy or if the patient was receiving treatment for the above condition. Patients who had insufficient data in their files regarding the presence of neuropathy and retinopathy received phone calls asking them if they have had symptoms of neuropathy or retinopathy or if they had laser treatment in the case of retinopathy.

### 3.3. Statistical Analysis

Data were entered and analyzed using the Statistical Package for Social Science (SPSS version 17). The overall prevalence of hypomagnesaemia was calculated and also in the subgroups defined by relevant variables. The bivariate association between hypomagnesaemia and a number of variables was assessed for statistical significance using the chi square test. Multivariate logistic regression was used to assess the independent effect of a given variable after adjusting for potential confounders. A P-value of < 0.05 was considered statistically significant.

## 4. Results

Participant’s characteristics: this study included 1105 diabetic patients (573 (51.9%) females and 532 (48.1%) males) aged between 24-86 years with a mean age (SD) of 57.1 (10.3) years. The demographic, anthropometric and clinical characteristics of the study population are presented in tables 1 and 2 respectively. In nearly 40% the length of diabetes was less than < 5 years while in 36% duration of diabetes was ≥ 10 years. Overall, 23% of the patients aged less than 50 years, while 42% of them were above or equal to the age of 60. Most of the diabetics in this study (99%) were either obese or overweight, therefore obesity was not included in further analysis because of lack of enough number of subjects with normal weight, and the mean (SD) BMI for the study participants was 33.2 (14.8). Seventy four perecent of the studied population were nonsmokers while 17% were current smokers. Seventy one perecent of diabetic patients were hypertensive, 4% were on loop diuretics, 21% were on thiazide diuretics for treatment of hypertension, 75% had dyslipidemia, 23% had retinopathy and 45% had neuropathy. Thirty six perecenthad controlled HbA1c < 7%, 27% had HbA1c between 7-7.9%, 17% had HbA1c between 8-8.9% while 20% of them had HbA1c ≥ 9%. Fifty nine perecent of diabetic patients involved in this study were on statin treatment for correction of dyslipidemia. The mean (SD) fasting blood sugar level for the study population was 178.1 (81.7) mg/dL, while the mean (SD) serum creatinine was 0.8 (0.6) mg/dL.

**Table 1.** Sociodemographic and Anthropometric Characteristics of the Study Participants (N= 1105)

Variable	No. (%)
**Gender**	-
Female	573 (51.9)
Male	532 (48.1)
**Age, y, mean ± SD= 57.1 ± 10.3**	-
<50	52 (22.8)
50-59	384 (34.8)
≥ 60	469 (42.4)
**Duration of Diabetes, y, mean±SD = 5.1 ± 4.8**	-
<5	444 (40.2)
5-9	259 (23.4)
≥ 10	402 (36.4)
**Smoking**	-
Not smoker	813 (73.6)
Past smoker	110 (10.0)
Current smoker	182 (16.5)

**Table 2.** Clinical Characteristics of the Study Participants (N = 1105)

Variable	No. (%)
**Hypertension**	-
Yes	785 (71.0)
No	320 (29.0)
**Dyslipidemia**	-
Yes	832 (75.3)
No	273 (24.7)
**Neuropathy**	-
Yes	496 (44.9)
No	609 (55.1)
**Retinopathy**	-
Yes	254 (23.0)
No	851 (77.0)
**HbA1c, %, mean ± SD= 7.9 ± 2.7**	-
< 7	393 (35.6)
7-7.9	298 (27.0)
8-8.9	191 (17.3)
≥ 9	223 (20.2)
**Patients on loop diuretics**	-
Yes	48 (4.3)
No	1057 (95.7)
**Patients on thiazide diuretics**	-
Yes	235 (21.3)
No	870 (78.7)
**Patients on statin**	-
Yes	652 (59)
No	453 (41)

### 4.1. Prevalence of Hypomagnesaemia in the Study Population (Table 3)

The overall prevalence of hypomagnesaemia in the study population (1105) was 19% (n = 210) (95% confidence interval 16.8%-21.4%). Hypertensive patients had significantly higher prevalence of hypomagnesaemia than normotensives (P-value 0.000001). On the other hand, 21% of patients with dyslipidemia had Hypomagnesaemia (P-value 0. 022).

Hypomagnesaemia was more prevalent in females (25%) in comparison to males (12%) in our study (P-value 0.000000). Hypomagnesaemia existed in 13% of patients with HbA1c < 7, 25% of those with HbA1c between 7-7.9%, 19% of those with HbA1c between 8-8.9% and 22% of those with HbA1c ≥ 9 (P-value 0.001).

In this study, hypomagnesaemia was significantly associated with increasing duration of diabetes, patients who had diabetes between 5-9 years or ≥ 10 years had 24% and 23% prevalence of hypomagnesaemia, respectively; while those who had their diabetes < 5 years had a prevalence rate of only 12% (P-value = 0.000011).

One third of patients on loop diuretics were hypomagnesaemic (P-value = 0.010), 25% of patients taking thiazide diuretic were hypomagnesaemic (P-value = 0.007) while 23% of patients on statin therapy were hypomagnesaemic (P-value = 0.000059).On the other hand, no association was found between the hypomagnesaemia and age, neuropathy or retinopathy.

**Table 3.** Prevalence of Hypomagnesaemia in Patients with Type 2 Diabetes according to the Relevant Demographic and Clinical Characteristics (N = 1105)

Variable	Hypomagnesaemia, No. (%)	P Value
**Age, y**	-	0.109
<50	37 (14.7)	-
50-59	74 (19.3)	-
≥ 60	99 (21.1)	-
**Gender**	-	0.000001
Male	65 (12.2)	-
Female	145 (25.3)	-
**Smoking**	-	0.002
Non	171 (21.0)	-
Past	19(17.3)	-
Current	20 (11.0)	-
**Duration of Diabetes, y**	-	0.00001
<5	53 (11.9)	-
5-9	63 (24.3)	-
≥ 10	94 (23.4)	-
**Hypertension**	-	0. 000001
Yes	176 (22.4)	
No	34 (10.6)	
**Dyslipidemia**	-	0.022
Yes	171 (20.6)	-
No	39 (14.3)	-
**Retinopathy**	-	0.220
Yes	55 (21.7)	-
No	155 (18.2)	-
**Neuropathy**	-	0.263
Yes	87 (17.5)	-
No	123 (20.2)	-
**HbA1c, %**	-	0.001
< 7	51 (13.0)	-
7-7.9	73 (24.5)	-
8-8.9	37 (19.4)	-
≥ 9	49 (22.0)	-
**Patients on loop diuretics**	-	0.010
Yes	16 (33.3)	-
No	194 (18.4)	-
**Patients on thiazide diuretics**	-	0.007
Yes	59 (25.1)	-
No	151 (17.4)	-
**Patients on statin**	-	0.000059
Yes	149 (22.9)	-
No	61 (13.5)	-

### 4.2. Multivariate Analysis of Factors Associated with Hypomagnesaemia 

In the bivariate analysis, hypomagnesaemia was significantly associated with female gender, dyslipidemia, hypertension, duration of diabetes, HbA1c level, smoking, diuretic and statin. After using stepwise logistic regression analysis, the only variables that remained significantly associated with hypomagnesaemia were female gender, hypertension, statin therapy, HbA1c level and the duration of diabetes (Table 4).

Female patients were found to be 2.4 times more likely to have hypomagnesaemia than male patients. Hypertensive patients were 1.8 times more likely to have hypomagnesaemia than those who were not hypertensive. Patients on statin therapy were 1.6 times more likely to develop hypomagnesaemia in comparison to those who were not on statins. Hypomagnesaemia was also significantly associated with HbA1c level. Patients who had HbA1c between 7-7.9% were 1.9 times more likely to have hypomagnesaemia in comparison to those with HbA1c < 7%, while those with HbA1c ≥ 9% were 1.8 times more at risk of having hypomagnesaemia compared to those with HbA1c < 7%. Furthermore, those who had a duration of diabetes between 5-9 years were two times more likely to have hypomagnesaemia compared to those with diabetes duration less than five years, while those with diabetes duration ≥ 10 years were 1.7 times more at risk of having hypomagnesaemia in comparison to those with diabetes duration less than five years after controlling for other variables.

Comparison between the present study and the National Vitamin D study with respect to prevalence of hypomagnesaemia: We compared the prevalence of hypomagnesaemia observed in this study with that observed in 3600 non-diabetic subjects who participated in the population –based National Vitamin D Study conducted in 2009. Hypomagnesaemia was much higher in the present study (19%) (95% CI, 16.8%-21.4%) than in the National Study (0.7%) (95% CI, 0.48%-1.07%) with an odds ratio of 32 (95%CI, 21-48.2).

On further analysis, when we categorized the data according to gender, hypertension and HbA1c ≥ 9% remained significantly correlated with the occurrence of hypomagnesaemia in males. On the other hand, HbA1c values between 7-7.9%, having a coronary artery disease (CAD), diabetes duration more than five years, and statin therapy were all significantly associated with hypomagnesaemia in females.

**Table 4.** Factors Independently Related to Hypomagnesaemia among Type 2 Diabetic Patients Using Multivariate Logistic Regression ^a^

Variable	OR	95% C.I.
**Gender**	-	-
Male	1	-
Female	2.42	(1.7-3.4)
**Hypertension**	-	-
No.	1	-
Yes	1.84	-
**HbA1c, %**	-	-
< 7	1	-
7-7.9	1.92	(1.4-3.2)
8-8.9	1.29	(0.9-2.3)
≥ 9	1.84	(1.2-3.1)
**Patients on statin**		-
No.	1	-
Yes	1.56	(1.1-2.2)
**Duration of Diabetes, y**		-
< 5	1	-
5-9	2.09	(1.2-4.0)
≥ 10	1.71	(1.2-4.4)

^a^ Abbreviations: OR: Odd's Ratio, CI: Confidence Interval

## 5. Discussion

In the present study low serum magnesium level among type 2 diabetic patients attending the (NCDEG) was found to be 19% (95% CI, 16.8%-21.4%) which was much higher than that observed among non-diabetics in the population-based National vitamin D study (0.7%) (95% CI, 0.48%-1.07%). In another population based study conducted in Iran the prevalence of hypomagnesaemia was much higher than the results found in the Jordanian National Vitamin D Study (4.6% vs. 0.7%), respectively (15). Higher prevalence rate of hypomagnesaemia in diabetic patients was also reported by other studies. For instance, a cross-sectional study conducted by Seyoum et al.(16) included a total of 159 subjects (44 patients with type 1 DM, 69 patients with type 2 DM and 46 non diabetic control) to assess the prevalence of hypomagnesaemia in Ethiopian patients with type-1 and type-2 DM. The study revealed that hypomagnesaemia was present in 65% of patients with diabetes. In other studies, hypomagnesaemia has been shown to occur in 25-38% (6, 17-19) of patients with diabetes, especially in those without good metabolic control. This wide range of difference in the prevalence between our study and other studies might be due to differences in the definition of hypomagnesaemia, techniques of magnesium measurements, and heterogeneity of the selected patient populations.

The mechanisms responsible for hypomagnesaemia in diabetic patients are not fully understood. Osmotic diuresis obviously accounts for a portion of magnesium loss, however magnesium intake may also play a role in magnesium deficiency. Suppressed levels of intracellular magnesium has been reported in patients with diabetes, and it has been suggested that circulating blood glucose independent of insulin levels, is a physiologic determinant of cellular ion hemostasis, suppressing intracellular free magnesium. Additionally, in patients with insulin resistance, hyperinsulinemia itself might contribute to magnesium depletion (7). Hypomagnesaemia was more prevalent in females in our study (25%) compared to males (12%). On further analysis, we found factors that could affect the prevalence of hypomagnesaemia are different among men and women, and we have no explanation for these gender differences, thus further studies are certainly indicated. Other studies conducted by Ascaso J F et al. (20) and Sheehan J P et al. (21) have also reported a higher prevalence of hypomagnesaemia in women compared to men at a 2:1 ratio.

Our study had shown that the rate of hypomagnesaemia was generally increased with increasing HbA1c from only 13% of patients with HbA1c < 7%, to 25% of those with HbA1c between 7-7.9%, 19% of those with HbA1c between 8-8.9% and 22% of those with HbA1c ≥ 9% (P-value = 0.001). This study also showed that hypomagnesaemia was significantly associated with increasing duration of diabetes: patients who had diabetes between 5-9 years or ≥ 10 years had a prevalence rate of hypomagnesaemia of 24% and 23%, respectively, compared to only 12% of those in whom diabetes duration was < 5 years. Our findings are consistent with the findings of Shaikh et al. (22) who evaluated the frequency of hypomagnesaemia in patients with type 1 and type2 DM. A Total of 100 diabetic patients were studied (77 with type 2DM and 23 with type 1DM). Hypomagnesaemia was identified in 8 (14.5%) of patients with type 1 diabetes and 47 (85.5%) of patients with type 2 diabetes. Of 55 hypomagnesaemic diabetic patients the Hemoglobin A1c (HbA1c) was raised in 40 (72.7%) patients. Shaikh et al.(22) also found that hypomagnesaemia was mostly prevalent in those who had diabetes duration between 6-10 years and 11-15 years (prevalence rate was 71% and 72%, respectively) compared to only 36% of patients with diabetes duration between 3 to 5 years.

A close relationship between metabolic control and hypomagnesaemia was confirmed by Fujii et al. (23), who found that hypomagnesaemia was particularly present in diabetic patients with advanced retinopathy and uncontrolled diabetes. However, no significant association was noticed between hypomagnesaemia and diabetes complications in our study. Our findings are also in contrast to the finding of Devalk et al. (24), who supported the association between hypomagnesaemia and progression of retinopathy in diabetic patients using insulin. Additionally, McNair et al. (25) also reported that retinopathy occurs more in magnesium deficient patients with insulin dependent diabetes mellitus (IDDM) and suggested hypomagnesaemia as a potential risk factor in the development and deterioration of diabetic retinopathy.

An important finding of this study is the significant association between hypomagnesaemia and hypertension which was independent from the potential confounding factors. Our data seem to support the Resnick’s hypothesis (26) suggesting that hypomagnesaemia in diabetic patients, which seems to be accentuated by the presence of hypertension, could explain the missing link between diabetes and hypertension.

An association between hypomagnesaemia and the use of lipid lowering agents was also noticed in our study. Such a finding is consistent with the findings of Haenni et al.(27) who reported that mean total serum magnesium concentration decreased following the treatment with Gimfibrazole and Simvastatin in patients with non-insulin dependent diabetes mellitus (NIDDM).

### 5.1. Strengths and Limitations of the Study

The main strengths of this study are its relatively large sample size (1105 patients), and the fact that it is the first ever study conducted in Jordan to assess the prevalence of hypomagnesaemia among type 2 diabetic patients. However, there are two main limitations. First, most of the participants were being treated with anti-diabetic and/or antihypertensive drugs which might have influenced the results. Second, only total serum magnesium levels were measured which does not take into the account the alterations in ionized Mg concentrations. 

### 5.2. Recommendation

The prevalence of hypomagnesaemia among patients with type 2 diabetes treated at the NCDEG was found to be 19% (CI, 16.8%-21.4%). Female gender, hypertension, statin therapy, HbA1c level between 7-7.9% or ≥ 9% and patients with duration of diabetes for more than five years were independently associated with hypomagnesaemia. We recommend periodic determination of magnesium level and appropriate magnesium replacement therapy particularly among the above defined groups.
